# FBXW7-mutated colorectal cancer cells exhibit aberrant expression of phosphorylated-p53 at Serine-15

**DOI:** 10.18632/oncotarget.3284

**Published:** 2015-03-16

**Authors:** Ningning Li, Federica Lorenzi, Eliana Kalakouti, Makhliyo Normatova, Roya Babaei-Jadidi, Ian Tomlinson, Abdolrahman S. Nateri

**Affiliations:** ^1^ Cancer Genetics & Stem Cell Group, Cancer Biology Unit, Division of Cancer and Stem Cells, School of Medicine, University of Nottingham, Nottingham NG7 2UH, UK; ^2^ Department of Neurodegenerative Disease, Institute of Neurology, University College London, London WC1N 3BG, UK; ^3^ Hillingdon Hospital, Uxbridge UB8 3NN, UK; ^4^ Molecular and Population Genetics Laboratory, the Wellcome Trust Centre for Human Genetics, University of Oxford, Oxford OX3 7BN, UK

**Keywords:** FBXW7, phopsho-P53(Ser15), colorectal cancer, drug resistance, CK1α

## Abstract

FBXW7 mutations occur in a variety of human cancers including colorectal cancer (CRC). Elucidating its mechanism of action has become crucial for cancer therapy; however, it is also complicated by the fact that FBXW7 can influence many pathways due to its role as an E3-ubiquitin ligase in proteasome degradation. FBXW7 and TP53 are tumour suppressors intensively implicated in colorectal carcinogenesis. Deletion mutations in these two genes in animal models mark the progression from adenoma to carcinoma. Although still largely unknown, the last defense mechanism against CRC at the molecular level could be through a synergistic effect of the two genes. The underlying mechanism requires further investigation. In our laboratory, we have used a phospho-kinase profiler array to illustrate a potential molecular link between FBXW7 and p53 in CRC cells. *In vitro* and *in vivo* assessments demonstrated aberrant induction of phosphorylated p53 at Serine 15 [phospho-p53(Ser15)] in human FBXW7-deficient CRC cells as compared to their FBXW7-wild-type counterparts. FBXW7 loss in HCT116 cells promoted resistance to oxaliplatin. Immunoblotting data further confirmed that reduction of phospho-p53(Ser15) may contribute to the decreased efficacy of therapy in FBXW7-mutated CRC cells. The findings may suggest the applicability of phospho-p53(Ser15) as an indicative marker of FBXW7-mutations. Phospho-p53(Ser15) regulation by FBXW7 E3-ligase activity could provide important clues for understanding FBXW7 behavior in tumour progression and grounds for its clinical applicability thereafter.

## INTRODUCTION

FBXW7 (F-box and WD repeat domain-containing 7, also known as FBW7, AGO, SEL10, CDC4) and TP53 are widely-known tumour suppressors and their genetic alterations lead to malignant transformation, metastatic spread and poor survival of cancer sufferers [1–4]. They are among the most commonly mutated genes after K-RAS and APC in colorectal cancer (CRC) [5–7]. FBXW7 constitutes one of the four subunits of SCF (SKP1-cullin-F-box)-E3 ubiquitin protein ligase complex, which functions in phosphorylation-dependent ubiquitination. To date, a wide array of SCF^FBXW7^ E3-ligase substrates have been identified and characterized, including cyclin E [8, 9], c-Myc [10, 11], c-Jun [12, 13], Notch [14], Presenilin [15], SREBP [16], Aurora-A [17], Krüppel-like factor 5 (KLF5) [18], DEK proto-oncogene [19], and peroxisome proliferator-activated receptor-γ coactivator-1α (PGC-1α) [20]. These and several other SCF^FBXW7^ E3-ligase substrates play central roles in cell division, growth, differentiation, cell-fate determination and maintenance of the phenotype of a variety of types of stem cells [3, 21–24]. There are several comprehensive reviews on FBXW7 functions as an E3-ligase in ubiquitin mediated proteasome-degradation [1, 3, 8–14].

Recent literature has reported the synergistic contribution of FBXW7 and TP53 to the suppression of gastrointestinal cancer [15, 16]. FBXW7 has been identified as a transcriptional target of TP53 and lower expression levels of FBXW7 upon TP53-mutations have been reported [15, 17, 18]. Parallel loss of the two tumour-suppressors was found to cooperate in tumourigenesis [16, 18, 19]. In line with such data, is the fact that most natural FBXW7 mutations in cancers are shown to exhibit a TP53 mutation [6, 7, 18]. Despite previous reports that FBXW7 is transcriptionally controlled by p53, little is known about their synergistic involvement in the molecular etiology of CRC. The p53-dependent relationship, reported in current literature, does not fully reveal the interplay between p53 and FBXW7, and p53 activity modulated by FBXW7-dependent mechanism(s) is yet unidentified.

Considering the multiple challenges to treatment response posed by a mutated tumour suppressor gene, elucidating FBXW7's mechanisms of action could be a breakthrough for cancer therapy. More recently, a number of groups demonstrated FBXW7-mediated drug resistance in human cancer cell lines *via* FBXW7-suppression and increased levels of pro-survival factor MCL1 and mTOR [20–23]. Wang et al. showed that loss of FBXW7 leads to rapamycin drug-resistant by inducing Epithelial-Mesenchymal Transition (EMT) in CRC cells [21]. However, it is still unclear whether this mechanism explains FBXW7 loss-conferred resistance to other standard chemotherapeutics such as 5-fluorouracil (5-FU), cisplatin and oxaliplatin.

Ultraviolet (UV) and DNA damage agents induced protein phosphorylation is one of the earliest events in modifying protein stability, and FBXW7 E3-ligase mediates the degradation of proteins in a phosphorylation-dependent manner [1, 3, 8, 24]. FBXW7 influences many pathways due to its role as an E3-ligase in proteasome-degradation. Loss of FBXW7 function is likely to result in failed regulation of its downstream targets and cellular acquisition of the hallmarks of cancer.

This study investigated the relationship between deregulation of FBXW7 E3-ligase activity and p53 phosphorylation. Our data show aberrant induction of phosphorylated-p53 at Serine 15 [phospho-p53(Ser15)] in human CRC cells that lacked FBXW7 as compared to their FBXW7 wild-type counterparts. TP53 is a key player in determining the response of colorectal cancer cells to oncogenic stress and chemotherapy by oxaliplatin and 5-FU [25]. UV-radiation but not oxaliplatin drug induced phospho-p53(Ser15) in CRC cells with FBXW7 deletion. Despite the accumulation of phospho-p53(Ser15) in mutant-FBXW7 CRC-tissues, FBXW7 does not directly interact with phospho-p53(Ser15) for degradation. Post-translational modification of p53 by its phosphorylation on Serine 15 has been one of the most extensively studied functional switch mechanisms in response to genotoxic stress. Serine15 residue of p53 is phosphorylated allowing p53 to be released from its normal physiological function [26, 27]. Subsequently, p53 stabilizes in the nucleus to act as a transcriptional activator for tumour suppression, implicating phospho-p53(Ser15) as a marker of FBXW7-associated carcinogenesis.

## RESULTS

### FBXW7 loss leads to induction of p53-phosphorylation at Serine-15

**Figure 1:**
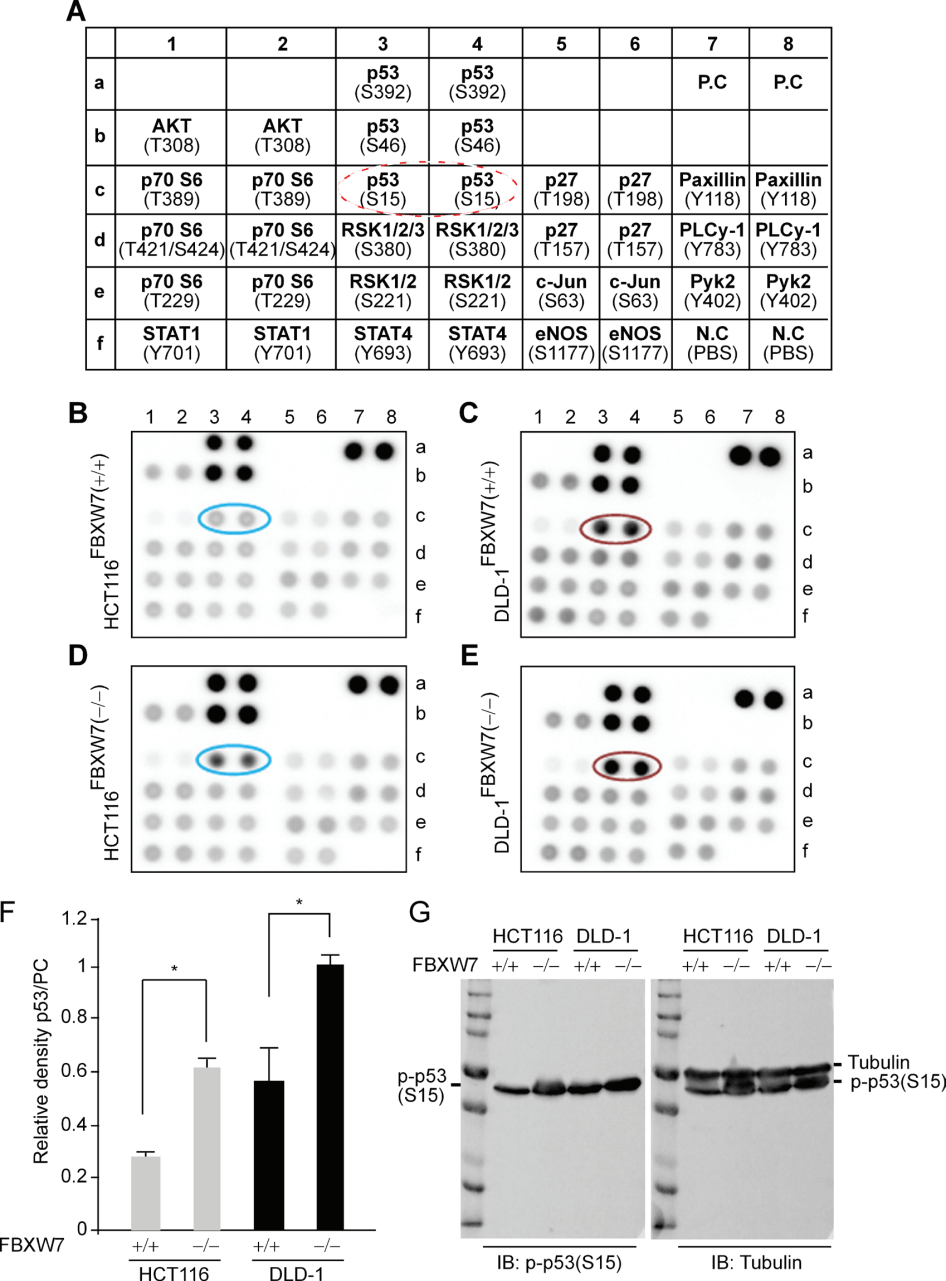
Human phospho-kinase-profiler-array (HPKPA) revealed induction of phospho-p53(Ser15) in *FBXW7*-null human CRC cells [FBXW7(−/−) vs. FBXW7(+/+)] **(A)** Schematic representation of the coordinates of the pre-coated phospho-specific antibodies in duplicate in a nitrocellulose-membrane. Dashed red-circle highlights the antibody position for phospho-p53(Ser15). **(B-E)** Outcome overview of the HPKPA using human CRC-cells expressing and lacking FBXW7; *FBXW7*-deficient HCT116 (D) and DLD-1 (E) cells [FBXW7(−/−)], compared to parental HCT116 (B) and DLD-1 (C) cells [FBXW7(+/+)]. **(F)** The expression of phospho-p53(Ser15) protein was significantly increased. Scanned values obtained using transillumination scanner of phospho-p53(Ser15) and Positive Controls (PCs) spots as corresponding to HCT116 and DLD-1 cell lines in B-E. The results shown are representative of experiments performed at three independent times. **(G)** Phospho-p53(Ser15) expressions was analyzed by Western blotting analysis. Protein extracts isolated from HCT116 and DLD-1 cell lines with FBXW7(−/−) and FBXW7(+/+) alleles were Western blotted for phospho-p53(Ser15) antibody and Tubulin (loading control).

Ablation of FBXW7 was shown to elevate the level of phosphorylated-substrate protein and its downstream signaling proteins. Such a phenomenon could inform about the disease mechanisms of colorectal carcinogenesis and the cellular pathways affected by homeostatic deregulation caused by an FBXW7 mutation. Post-translational modification of p53 by phosphorylation may be an important mechanism underlying regulation of p53 stabilization and function. However, the molecular and cellular mechanisms that link FBXW7 and p53 following phosphorylation are unclear. An *in vitro* human phospho-kinase array (HPKPA) with multiple p53-phosphoacceptor sites (Figure 1A), was used to assess changes to the protein phosphorylation profile. We and others have reported that HCT116 and DLD-1 cell-lines harboring wild-type FBXW7; *FBXW*7(+/+), or inactivated FBXW7; *FBXW*7(−/−), have the characteristics of human CRC cells without or with FBXW7-mutation [19, 20, 23, 28]. In particular, they provide an excellent *in vitro* model to delineate the molecular mechanisms that contribute to neoplasia. Remarkably, in the absence of FBXW7, both HCT116 and DLD-1 showed a substantial increase in p53 phosphorylation at Serine-15 as compared to control cells (Figure 1F), while phosphorylation at Serine-46 and Serine-392 remain unchanged (Figure 1, 1B vs. 1D and 1C vs. 1E). Western blot analysis showed an increase of p53 phosphorylated at Ser-15 in *FBXW*7(−/−) versus wild-type *FBXW*7(+/+) (Figure 1G).

**Figure 2:**
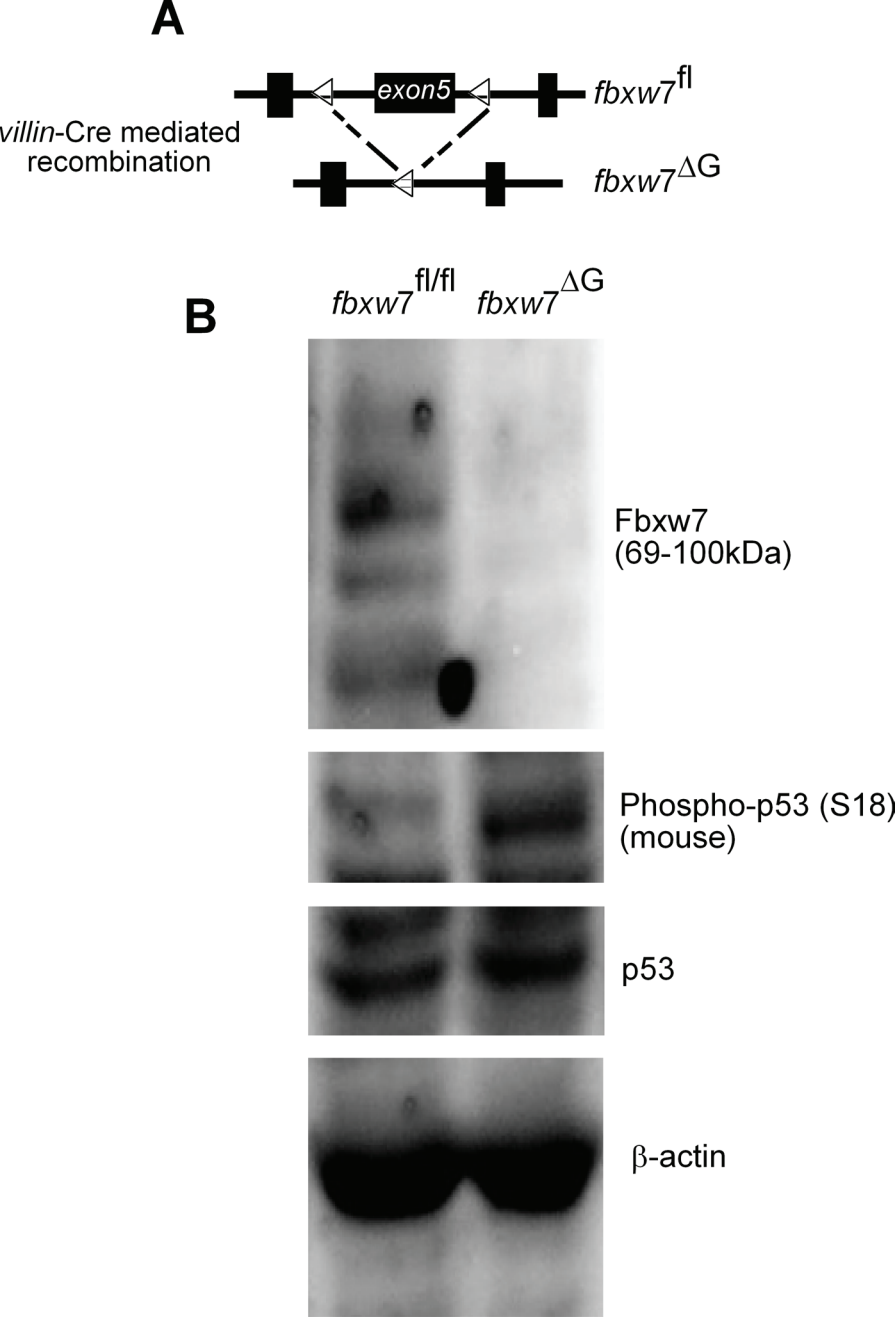
Induction in the level of the of murine phospho-p53(Ser18) in the intestine of fbxw7^ΔG^ mice **(A)** Schematic shows the floxed Fbxw7 allele (*fbxw*7^fl/fl^) before and after *villin*-Cre recombination to generate gut-specific conditional Fbxw7 inactivation (*fbxw*7^ΔG^) mice. **(B)** Western blot analysis of *fbxw*7^fl/fl^ and *fbxw*7^ΔG^ intestinal proteins using antibodies against Fbxw7, p53, phospho-p53(Ser15), and the loading control β-actin. Experiments were performed on at least two independent occasions.

The Ser15 phosphorylation site is evolutionarily conserved and corresponds to Ser18 of murine p53, which has not been as extensively examined as that of human p53. Mice harbouring an *fbxw*7 allele in which exon5 was flanked by two loxP sites were crossed to *villin*-cre transgenic mice as previously described [28, 29] (Figure 2A). We examined p53 phosphorylation in mouse intestinal tissues harboring *fbxw*7 deletion. We detected enhanced phosphorylation of p53 at Serine-18 with the phospho-specific p53 (Ser15) antibody [30]. Phosphorylation of Serine-18 regulates distinct p53 functions in mice, including p53-dependent transcripton, apoptosis, and cell cycle arrest after DNA damage [30]. There was accumulation of phosphorylated p53 at Serine-18 but despite the high levels of phospho-p53(Ser18), there was only a very small increase in total levels of p53 (Figure 2B).

### FBXW7-dependent induction of phospho-p53(Ser15) in CRC cells treated with UV radiation

**Figure 3:**
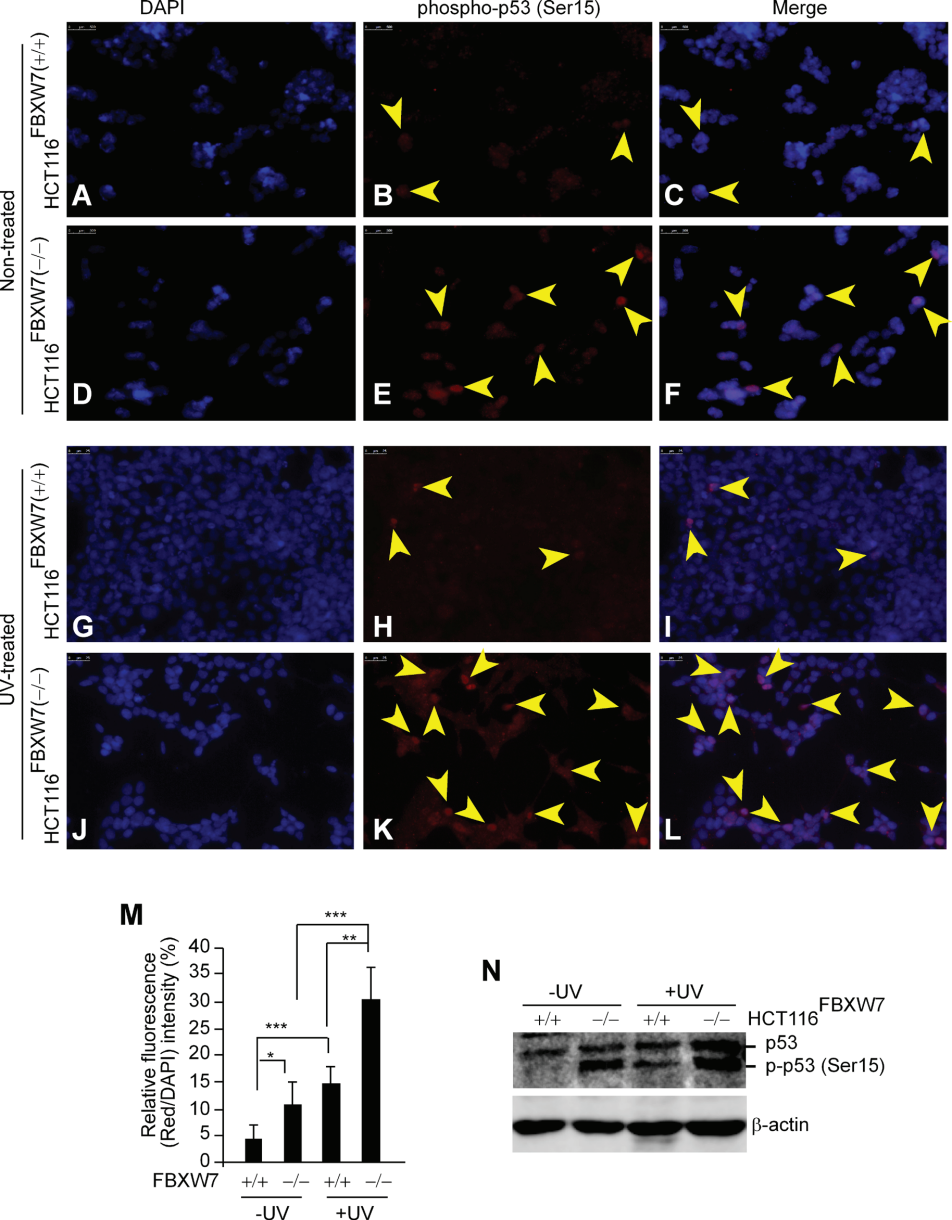
*In vitro* validation of phospho-p53(Ser15) accumulation in *FBXW7*-deficient human CRC cells **(A-L)** Immunofluorescent staining of phospho-p53(Ser15) protein (red) in non-treated (A-F) and 50 kJ/m^2^ UV-treated (G-L) HCT116 cells. UV stimulated induction of phospho-p53(Ser15) protein in general (H & K). In both conditions, *FBXW7-*deficient cells (E & K) obtain higher expression levels of phospho-p53(Ser15) than the corresponding control cells (B & F). Nuclei were visualized with 4′, 6-diamidino-2-phenylindole (DAPI)-blue fluorescent stain (A, D, G, and J). Overlaid images (C, F, I, and L) showed nuclear localization of phospho-p53(Ser15). Arrows indicate phospho-p53(Ser15) distribution. Bars, 25 μm. Figures are representative of duplicate experiments performed on three separate occasions. **(M)** Quantification analysis of fluorescence intensity of IF images. **(N)** Western blotting analysis of phospho-p53(Ser15) expression; untreated and exposed to 50 kJ/m^2^ UV. β-actin served as a loading control. All experiments were repeated at least three times.

FBXW7 is involved in the control of genetic instability [16, 19]. A strong association has been found between phosphorylation of p53 on Serine-15 and exposure to UV irradiation [31, 32]. In order to evaluate phospho-p53(Ser15) induction's dependence on FBXW7 in UV-treated CRC cells, we used HCT116 cells harboring wild-type and inactivated FBXW7. Immunofluorescence (IF) staining showed nuclear phospho-p53(Ser15) was almost undetectable in control HCT116^FBXW7(+/+)^ cells, but was markedly increased in FBXW7-null HCT116^FBXW7(−/−)^ cells (Figure 3, 3E vs. 3B; 3F vs. 3C and 3M). This finding was supported by Western blot (WB) analysis (Figure 3N). In addition, we applied UV-treatment to the HCT116 cells to provide clearly distinguishable results. Treatment with UV-radiation enhanced phospho-p53(Ser15) induction in both the wild-type and FBXW7-mutated cells (Figure 3, 3H & 3K; 3M, right panel). Nonetheless, phospho-p53(Ser15) in HCT116^FBXW7(−/−)^ cells was markedly higher than that in HCT116^FBXW7(+/+)^ cells, with both cytoplasmic and nuclear localization (Figure 3, 3K vs. 3H; 3L vs. 3I). The WB data were consistent with the IF results (Figure 3, 3M and 3N). Our results validated the enrichment of phospho-p53(Ser15)-induction in FBXW7-deficient CRC cell lines. Subsequently we further investigated whether phospho-p53(Ser15) could provide diagnostic and/or prognostic value for CRC-biopsies with *FBXW*7-mutations.

### Phospho-p53(Ser15) protein accumulation in human CRC-tissues with FBXW7-mutation

**Figure 4:**
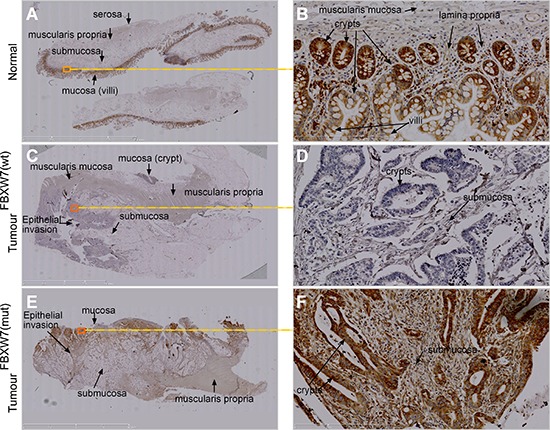
IHC validation of phospho-p53(Ser15) accumulation in *FBXW7*-mutant human CRC-tissues **(A-F)** Representative images of chromogenic IHC-staining of paraffin-embedded *FBXW*7*-*wild-type (wt) and -mutated (mut) CRC-tissue sections for phospho-p53(Ser15) expression. Specimens of healthy small bowel (*n* = 1) (A and B), *FBXW*7-wt CRC (C and D), and *FBXW*7-mut CRC (E and F) show enrichment, annihilation, and remarkable increment of phospho-p53(Ser15) expression. Bars, 50 μm (A, C, E); 100 μm (B, D, F).

**Figure 5:**
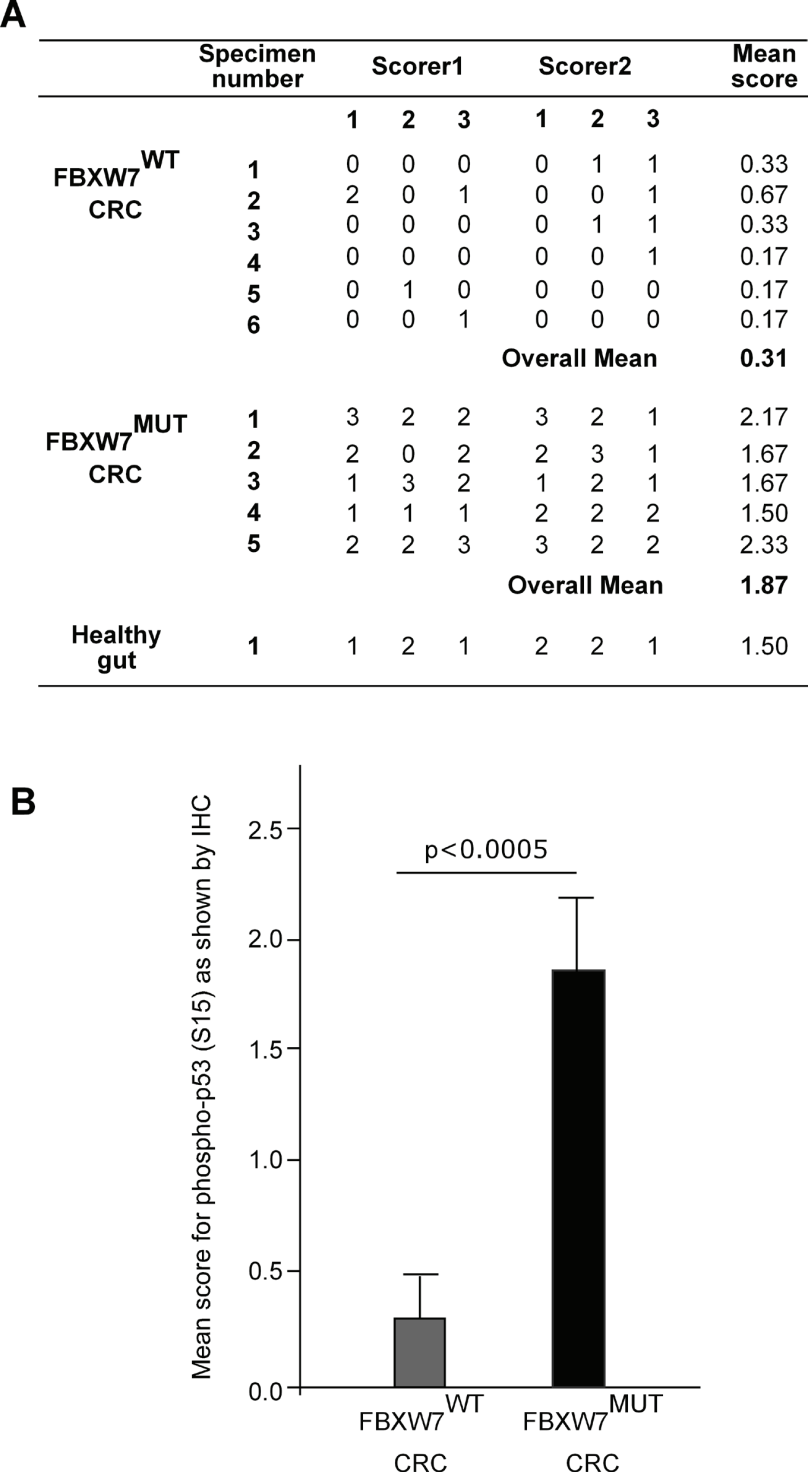
Phospho-p53(Ser15) expression scores in sections from CRC-tissues with and without *FBXW7*-mutations **(A)** IHC for human CRC-tissues evaluated semi-quantitatively by two independent observers within 3-microscopic views in the individual slides. Expression scale of the phospho-p53(Ser15) protein was determined by the intensity of the staining, and thus scored using an ordinal scale: 0 = no staining, 1 = lowest staining, 2 = intermediate staining and 3 = extensive staining. **(B)** *P* value (***p < 0.0005; mean ± S.D.; mean for FBXW7^WT^ group = 0.31 with S.D = 0.1945; and mean for FBXW7^Mut^ group = 1.87 with S.D. = 0.3600) was gained from Student's *t*-test comparing phospho-p53(Ser15) expression in CRC-tissues with and without *FBXW*7-mutations.

We carried out *in vivo* investigations to validate the phospho-p53(Ser15) induction in CRC tissues excised from patients with FBXW7-mutated tumours. Immunohistochemical (IHC) comparison of phospho-p53(Ser15) expression in wild-type and FBXW7-mutated human CRC-tissue was carried out. The intensity of the IHC staining observed in the two tissue types (wild-type vs. mutants for FBXW7), was evaluated in a semi-quantitative manner. In CRC tissues, the architecture of the gut wall was chaotic as expected for both cancer specimens: *FBXW*7- wild-type (control) and -mutant (Figure 4, 4C-4F). Irregular and distorted crypts were evident invading the submucosal area of CRC tissues (Figure 4E, 4F). Expression of phospho-p53(Ser15) protein was completely lost in *FBXW*7 wild-type tissue (Figure 4C, 4D) [mean staining-score; 0.33 (Figure 5A)], irrespective of the tumour-type or the presence of p53-mutation. On the contrary, *FBXW*7-mutant CRC showed substantial of phospho-p53(Ser15) induction in the mucosa and early submucosal invasion (Figure 4E, 4F) [mean staining-score; 1.87 (Figure 5A)]. Healthy human small bowel tissue, which was available as an additional control, exhibited a consistent distribution of phospho-p53(Ser15) protein in the mucosal lining of the structured bowel wall architecture (Figure 4A, 4B) [mean staining-score; 1.50 (Figure 5A)]. In general, phospho-p53(Ser15) was enriched in the intestinal crypts, but was much less in the differentiated cell types residing in the villi (Figure 4B). This may suggest that phospho-p53(Ser15) could be detected in cancer and crypt-based cells which exhibit poor differentiation in contrast to the cells of the villi. Collectively, compared to the negligible expression in control CRC tissues, remarkable accumulation of phospho-p53(Ser15) protein was observed in *FBXW*7-mutant samples.

**Figure 6:**
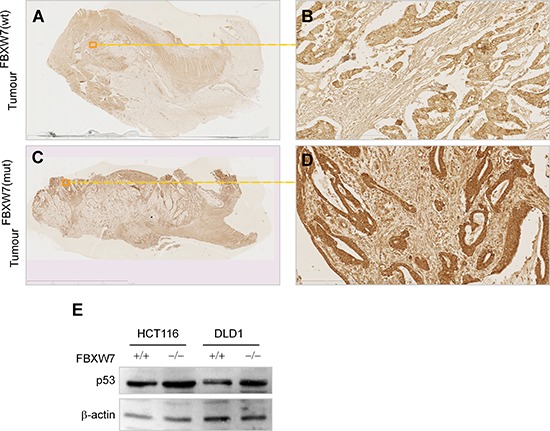
p53 levels remained unchanged in *FBXW7*-mutant human CRC-tissues **(A-D)** IHC for p53 in human CRC with (C, D) and without (A, B) FBXW7 mutations. Boxed line indicates magnified tissue area. Scale bars; 50 μm. **(E)** Western blotting analysis of p53 expression in HCT116 and DLD-1 cell lines expressing or lacking FBXW7. β-actin was blotted for loading control. All experiments were repeated for two times.

Even though TP53 mutations have been reported in over 50% of human tumours [33], it was not possible to profile the TP53 status by molecular methods in the CRC-tissues utilized in our previous studies (Ian Tomlinson, personal communication) [28, 29]. However, our data show heterogeneous pattern of p53-staining. There was more p53 staining at the invasion front of CRCs with a small increase (not statistically significant) in total levels of p53 (*p* = 0.072) among CRC tissues with *FBXW*7-mutation (Figure 6, 6A-6E & data not shown). Of interest was the fact that the only controlled variable for the wild-type CRC-tissues examined was the presence of intact FBXW7. Similarly, the only controlled variable for the *FBXW*7-mutated tissues was the presence of an *FBXW*7 mutation with other cancer mutations remaining unidentified. Regardless of the unknown nature of the non-*FBXW*7 mutations, the presence of an *FBXW*7-mutation alone was adequate to give a consistent pattern of phospho-p53(Ser15) induction (mean-range; 1.67–2.33). Statistical analysis confirmed that phopsho-p53(Ser15)-induction in *FBXW*7-mutant tissues was highly significant (*p* < 0.0005) (Figure 5B). Although only a limited number of samples was available, the results pointed towards the suitability of phospho-p53(Ser15) induction as an independent indicative marker of CRC with *FBXW*7-mutation.

### Loss of FBXW7 leads to oxaliplatin drug resistance in CRC cells through differential expressions of the TP53 family of transcription factors

**Figure 7:**
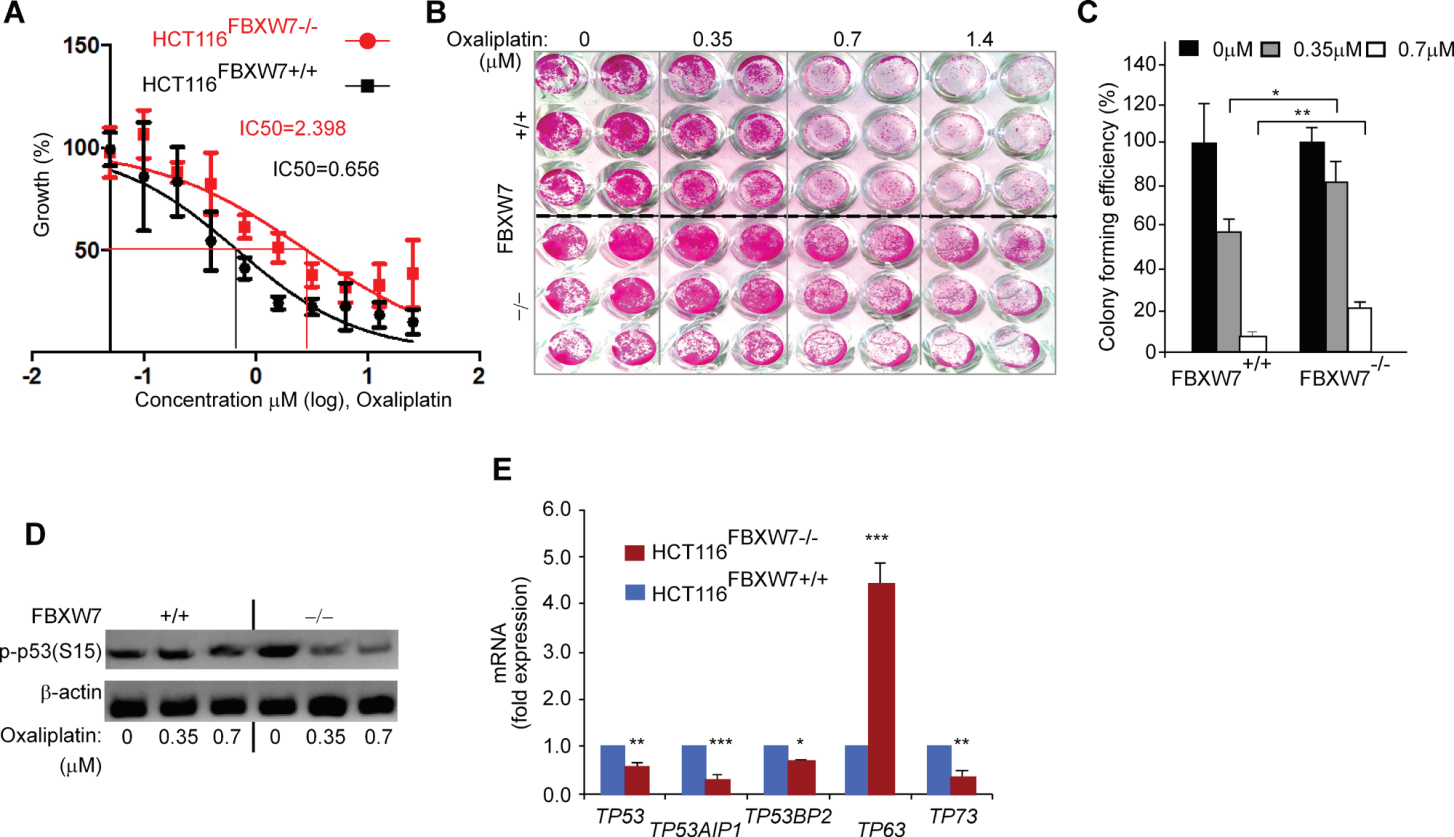
FBXW7 deficiency induces oxaliplatin drug resistance property of HCT116 CRC cells After starvation overnight, synchronised HCT116^FBXW7(+/+)^ and HCT116^FBXW7(−/−)^ cells were treated with increasing concentrations of the oxaliplatin; for 72 h, IC50 **(A)** and cell viability **(B)** determined using SRB at 540 nm in a microplate reader. **(C)** An increased colony forming efficiency of FBXW7–/– HCT116 cells compared with parental FBXW7+/+ HCT116 cells and graphs show mean ± SEM of assay data, representative of six replicates in three independent experiments (*P < 0.05, **P < 0.01, ***P < 0.001). **(D)** phospho-p53(Ser15) protein expression measured at low (0.35) and IC50 dose (0.7 μM) oxaliplatin concentration in HCT116^FBXW7(+/+)^ and HCT116^FBXW7(−/−)^ cells by Western blotting. **(E)** p53-mediated genes expression was determined by qRT-PCR in HCT116^FBXW7(−/−)^ cells (red), first normalized to GAPDH followed by normalization to HCT116^FBXW7(+/+)^ cells (blue). Data are mean ± SEM (*P ≤ 0.05; **P < 0.01; ***P < 0.001). Experiments were performed in triplicate for each genotype and repeated at least on three independent occasions.

To evaluate the cytotoxicity of oxaliplatin in CRC-cells harboring *FBXW*7-deletion, both HCT116^FBXW7(+/+)^ and HCT116^FBXW7(−/−)^ cells were treated with increasing concentration of oxaliplatin (0.05 to 25.6 μM), separately, for 3 days. SRB assays were performed, dose-response curves generated and IC50 values calculated. The assay was carried out in triplicate and repeated at least three times. The Student's *t*-test was used to value the statistical-significance. The results showed that FBXW7-mutated HCT116^FBXW7(−/−)^ cells conferred resistance to oxaliplatin drug (Figure 7, 7A-7C). IC50 of HCT116^FBXW7(−/−)^ for oxaliplatin was almost four times higher than the IC50 of the corresponding *FBXW*7-wild-type cell line. Indeed, the IC50 of HCT116^FBXW7(+/+)^ cells for oxaliplatin was about 0.6567 μM and that of HCT116^FBXW7−/−^ cells was about 2.398 μM (Figure 7A). The results show that *FBXW*7-deleted cells demonstrated higher tolerance to the effects of oxaliplatin drug (Figure 7, 7B, 7C & data not shown). IC50 values for control HCT116 cells with wild-type FBXW7 are consistent with previously reported values for the same drugs [34]. The data suggest that FBXW7 loss confers HCT116 cells resistance to oxaliplatin.

To address the hypothesis of an existing relationship between mutated-FBXW7 and phopsho-p53(Ser15) protein in response to drug treatment, the expression level of phopsho-p53(Ser15) protein was assessed by Western blotting (Figure 7D). In contrast to UV-irradiation, Western blotting results showed that expression of phospho-p53(Ser15) decreased after oxaliplatin treatment (Figure 7D) in HCT116^FBXW7−/−^ cells. Hence, the decreased phospho-p53(Ser15) on its own might not affect the cell's ability to induce apoptosis. To assess the impact of loss of FBXW7 and TP53-mediated gene expression on HCT116^FBXW7(−/−)^ cells behavior, mRNA was isolated from equal numbers of HCT116^FBXW7(+/+)^ and HCT116^FBXW7(−/−)^ cells (Figure S1). The differential expression of members of the *TP53/TP63/TP73* family of transcription factors and apoptosis-related TP53-mediated gene expression (*TP53AIP1* and *TP53BP2*) was analyzed by real-time-PCR [35] (Table S1). The analysis highlights significant up-regulation of *TP*63 in HCT116^FBXW7(−/−)^ cells while both *TP*53 and *TP*73 expression appeared to be down-regulated (Figure 7E). Apoptosis-stimulating protein of TP53AIP1 and TP53BP2 expression was simultaneously down-regulated (Figure 7E). The result may suggest that induction of phopsho-p53(Ser15) by loss of SCF^FBXW7^-E3-ligase activity could contribute to other pathways function to block the p53 activation [27] and consequently decreased efficacy of therapy in chemo-resistant CRCs.

### Phospho-p53(Ser15) may not be a direct target of SCF^FBXW7^-E3 ubiquitin ligase

**Figure 8:**
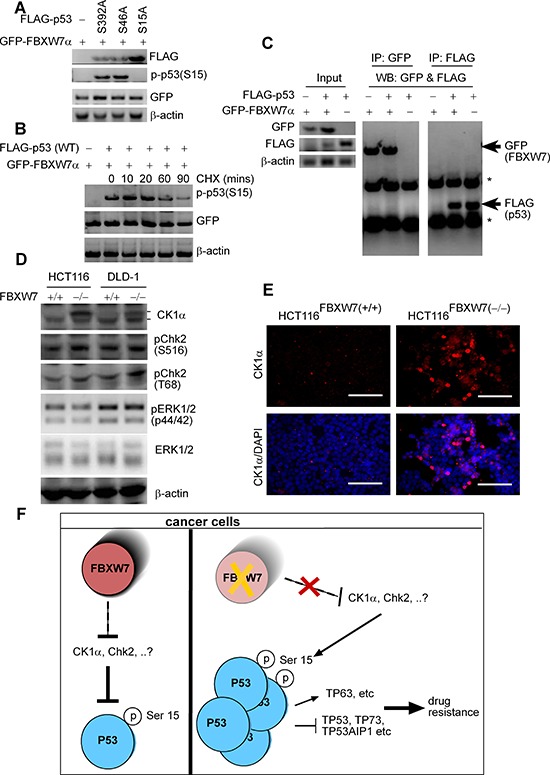
phospho-p53(Ser15) is regulated by FBXW7 but not through direct interaction **(A)** FLAG-tagged versions of the indicated p53 mutant and GFP-FBXW7 proteins were expressed in HEK293T cells, and lysates were subjected to immunoblotting with anti-FLAG, anti-p-p53(Ser15) and anti-GFP. β-actin was used as a loading control. **(B)** HEK293T cells were treated with cycloheximide (CHX) for the indicated time points. Lysates were examined by Western blotting anti-p-p53(Ser15) and anti-GFP. β-actin was used as a loading control. **(C)** Western blot analysis of whole-cell lysates (input) (left-panel) and immunoprecipitates (IP) with GFP (middle-panel) and FLAG-p53 (right-panel) derived from 293T cells transfected with GFP-FBXW7 together with the FLAG-p53 constructs. Thirty hours after transfection, cells were pretreated with 10 μM MG132 for 4 h to block the proteasome pathway before cell collection. Both IPs was probed by anti-GFP and anti-FLAG antibodies simultaneously. **(D)** HCT116 and DLD-1 cells lacking or expressing FBXW7 were subjected to immunoblotting with anti-CK1α, pChk2 (S516), pChk2 (T66), phospho-p44/42 MAPK (pERK1/2) and p44/42 MAPK (ERK1/2). β-actin was loaded as a loading control. All experiments were repeated at least three times. **(E)** Immunofluorescent staining of anti-CK1α of HCT116^FBXW7(+/+)^ versus HCT116^FBXW7(−/−)^ cells. **(F)** Schematic of molecular changes may occur in the HCT116^FBXW7(−/−)^ compared with parental HCT116^FBXW7(+/+)^ cells treated with the oxaliplatin.

In the present studies, we demonstrated through several lines of evidence that FBXW7 ablation in CRC cells results in the accumulation of phospho-p53(Ser15). The mechanism of this is still uncertain. We therefore studied the FBXW7/p53-correlation further to validate the phospho-p53(Ser15) phenomenon. Consistent with the HPKPA results, mutations at either Ser46 (S46A) or Ser392 (S392A) did not stabilize phospho-p53(Ser15) in the presence of FBXW7 (Figure 8A). Western blotting data with cells that overexpress the p53(S15A) mutant protein also determined the anti-phospho-p53(Ser15) antibody specificity (Figure 8A). Furthermore, co-expression of FBXW7 decreased the steady-state levels of p53(wt) (Figure 8B). We next examined whether the FBXW7 protein would bind to p53 protein and vice versa. Immunoprecipitation (PI) of either FLAG epitope-tagged p53 or GFP fused-FBXW7α showed no interaction between these proteins (Figure 8C). These data suggest that FBXW7 does not act directly, but indirectly regulates the abundance of phospho-p53(Ser15).

Furthermore, high levels of phosphorylation of p53 at Serine-15(Ser15), modulate cell cycle checkpoint kinase Ataxia-Telangiectasia Mutated (ATM) and Ataxia-Telangiectasia and Rad3-related (ATR) that impede MDM2 binding to p53 and subsequently promote p53 accumulation and activation in response to DNA damage [32, 36]. Phosphorylation of Serine 15 is considered to be an initiating and nucleating event in p53 activation, especially in combination with Ser20 and Thr18 phosphorylation. ATM and ATR kinase however, in turn, activates the effector kinase such Chk2 and ERK to phosphorylate Ser15, along with several other residues in p53 [37–39]. Following p53 Ser15 phosphorylation, protein kinase CK1 can also sequentially phosphorylate Thr18 using the phosphorylated Ser15 as a recognition determinant [40, 41]. Given these findings, we examined the level of these kinases in both HCT116 and DLD-1 cells lacking and expressing FBXW7. Both Chk2 phosphorylation at Ser516, and Thr68 protein levels were slightly increased in HCT116(−/−) and DLD-1(−/−) cells compared with control (+/+) cells, whereas CK1α level remarkably increased, especially its long isoform (Figure 8D). Total (phosphorylated and non-phosphorylated) levels of ERK 1/2 remained unchanged (Figure 8D). CK1α has two alternatively spliced isoforms, one short (known as CK1αS) and one long (known as CK1αL). Both human CK1α isoforms contain a common 12-amino-acid short insert located at the carboxyl terminus, whilst CK1αL itself includes a 28-amino-acid long insert (i.e. encoded from exon 5 of CK1αL gene) located in the central kinase catalytic domain [42, 43]. Therefore, human CK1α isoforms are composed of 365 or 337 amino acids due to presence or absence of the exon 5. Consistent with Western blotting data, immunofluorescent staining showed remarkably increased expression levels of a nuclear splice form of CK1α (Figure 8E). Further analysis, beyond the current scope of this paper, would be required to validate the possibility that FBXW7 could modulate phospho-p53 via targeting CK1α for degradation.

## DISCUSSION

Our experiments do not point to phospho-p53(Ser15) as a direct binding substrate for FBXW7 E3-ubiquitin ligase degradation. Cellular stress generated by accumulation of FBXW7-substrates (for example, Cyclin E and c-Myc) may induce the TP53 pathway via phospho-p53(Ser15) induction [44, 45]. However, in the colon/intestine, we and others have shown that expression of c-Myc and Cyclin E were unaffected by loss of intestinal/colonic-*fbxw*7 [18, 28, 29, 46]. This could suggest a potential route for FBXW7 regulating phosphorylated p53 protein expression through controlling upstream kinases. Considering the fact that CK1α is an essential regulator of p53 phosphorylation [40, 41, 47], this study provides the possibility that FBXW7 could modulate phospho-p53 via targeting CK1α for degradation. Therefore, it could be postulated that loss of FBXW7 could result in CK1α accrual *in vivo*, which subsequently leads to increased p53 stabilization and activation. Indeed, we and other recently demonstrated that neither FBXW7 abolishment nor CK1α deletion *in vivo* caused immediate intestinal tumour formation [28, 29, 48], possibly due to compensation from the p53 pathway [16, 48]. Furthermore, we have previously shown an up-regulation of β-catenin in HCT116(FBXW7−/−) cells [28]. β-catenin is phosphorylated by CK1 (EMBO J. 2002 Apr 2;21(7):1733–42). Moreover, CK1α role in malignancy is still ambiguous as it was previously reported as an oncogene [49] and it may also function as a tumour suppressor gene [50]. There are, as yet, no *in vivo* or *in vitro* data showing a direct link between CK1α. and p53 Ser15. On the other hand, our data suggest that FBXW7 could be an upstream regulator of p53 and CK1α. Considering the possibility that CK1α might act as an important co-effector protein linking FBXW7 and other cellular pathways, such as Wnt and p53, in this respect, we may hypothesis a synergistic role for FBXW7 and p53 in suppression of intestinal tumourigenicity and metastasis. Moreover, of interest, are the observations that suggest that wild-type TP53 in tumours may not have the same transcriptional activity often found in their non-transformed counterpart tissues [51]. Hence, further studies are required to prove a possible association between CK1α and p53(Ser15), and provide significant evidence for CK1α-mediated up-regulation phospho-p53(Ser15) in tumours with FBXW7 mutations. Furthermore, these data may also prompt us to reconsider the status of p53 phosphorylation in relation to expression of different isoforms of CK1α kinase. This may be explained by its diverse roles in different cellular processes [52, 53] and by the distinct functions and subcellular locations of its two splicing variants.

Taken together, the results from cytotoxicity and viability assays suggest that loss of FBXW7 function can confer resistance to DNA-damage agent oxaliplatin in CRC cells. FBXW7 might act as a core factor in different mechanisms used by cancer cells to become resistant to chemotherapeutic drugs. Determining these mechanisms of eluding DNA damaging agents, probably through the modulation of SCF^FBXW7^ protein substrate(s), may be crucial for understanding tumour development and its response to current chemotherapy. It has been shown that p53(Ser15) phosphorylation can stimulate p53 activity. In contrast, it was also found that phosphorylation of Serine-15 can block binding of p53 to transcription Factor II D, and inhibit the formation of a DNA promoter complex for transcription [27].

Widely-accepted evidence reveals that tumours exhibit a different type and substantially a greater degree of p53-phosphorylation than normal cells rendering in this way the tumour suppressor nonfunctional [51]. Recently, reports showed that p53 up-regulates FBXW7 [15, 17] whereas it represses ZEB family transcriptional regulator (ZEB1 and ZEB2), implicated in epithelial-mesenchymal transition (EMT) [54]. Western blotting and immunohistochemistry data did not exhibit statistically significant differences in total p53 protein levels in HCT116^FBXW7−/−^ cells and FBXW7-mutated CRC tissues, compared with parental HCT116 cells which have functional wild-type TP53, and FBXW7-wild-type CRC tissues (Figure 5). There is a lot of research activity as to whether, and how, TP53 family members interact or conflict with each other in apoptosis and tumour suppression [55]. Induction of death of neurons and mouse embryo fibroblasts by p53 requires coalescence of p63 and p73 [56], while not required for the induction of apoptosis in T cells [57]. DeltaNp63 isoform of p63 have been associated with transcriptional activation of ATM and p53 Serine-15 phosphorylation in immortalized human keratinocytes [58]. However, consistent with the recent published data [59], we did not observe any significant changes in levels of ATM kinase from protein extracts isolated from HCT116 and DLD-1 cell lines with FBXW7(−/−) and FBXW7(+/+) alleles in a WB analysis (data not shown). Whether FBXW7-mutation induces EMT via p53/p63/p73 regulation or vice versa, and provides a possible explanation for the prominent chemoresistance observed in CRC, remains to be elucidated.

Based on our results, further work to investigate the cellular mechanisms implicated in the phospho-p53(Ser15)/FBXW7 phenomenon are necessary to provide more insight into the underlying mechanisms. Following validation of the phospho-p53(Ser15) protein induction, an enquiry was raised whether such stimulation serves any function within the tumour other than its use as an indicative marker of FBXW7-mutations. Does the phospho-p53(Ser15) induction serve any role in the FBXW7-associated carcinogenesis due to a likely gain/loss/change of P53/P63/P73 function? Our current data as presented are limited to few tumour specimens. To assess if FBXW7 mutation confers resistance to oxaliplatin relevant to prognosis or treatment response, further studies should be carried out using a large number of patients treated with oxaliplatin.

## METHODS

### Human colorectal cancer (CRC) tissues

Paraffin-embedded human colorectal tumour tissue sections, wild-type (FBXW7^WT^ CRC) and positive for FBXW7-mutation (FBXW7^Mut^ CRC) were kindly provided by the molecular and population genetics laboratory, Wellcome Trust Centre, University of Oxford. The ethical and R&D approval for use of human tissue was obtained from the local research-ethics committee and Trust R&D office, in Oxford and Nottingham, as described previously [28].

### Human phospho-kinase profiler array (HPKPA)

In order to identify signaling pathways altered by FBXW7, a human phospho-kinase array kit (R&D Systems, UK) was used. This consists of a nitrocellulose membrane (Figure 1A) having control antibodies against phosphorylated proteins which are spotted in duplicate. It allows the detection of the relative levels of phosphorylation of protein/kinase phosphorylation sites (antibodies for those sites were selected and spotted in duplicate on nitrocellulose membrane).

Two CRC cell lines (HCT116 and DLD-1 cells) harboring wild-type-FBXW7; *FBXW*7(+/+), or inactivated *FBXW*7(−/−), were rinsed with PBS, solubilized in the Lysis Buffer supplied with kit and with Halt Protease and EDTA-free Phosphatase Inhibitor Cocktail (100X) was added as 1:100 concentration (Pierce, UK). The lysates were gently rocked at 4°C for 30 min and then centrifuged at 14, 000x g. The protein was quantified using the Bradford protein assay (Bio-Rad) and the four different cell lysates were adjusted to 100 μg in 1ml of array buffer supplied with the kit. After blocking the nitrocellulose membrane by rocking in Buffer 1 for 1 hour at room temperature, diluted lysate contain 100 μg protein was added and incubated on a rocking platform overnight at 4°C. Washing with wash buffer was followed by adding the diluted detection antibody cocktail to corresponding part of the membrane and incubation for 2 hours at room temperature on a rocking platform. Another wash was carried and the membranes were incubated with diluted Streptavidin-HRP for 30 min then followed by another washing. The spots were detected using enhanced chemiluminescence (ECL) using the FlourChem FC2 imaging system (Alpha Innotech).

### Immunohistochemistry (IHC) and immunofluorescence (IF)

Paraffin-embedded tissue sections were dewaxed, rehydrated as described [28, 60]. Peroxidase activity in tissue samples quenched with 1.6%H2O2 (5.33 ml of 30%H2O2 [Fisher] added in 94.67 ml of PBS) for 10 min, and sections washed with PBS for 5 min. The specimen region on each section slide circled with a wax PAP-pen, and incubated for 30 min in 100 μl Serum-blocking buffer/slide (10%[v/v] normal-serum and 1%[w/v] Bovine-Serum-Albumin (BSA) in PBS. The sections incubated in primary and biotinylated-secondary-antibody for 1 hr at RT, respectively, followed by PBS wash for 3 × 3 mins. Antibodies freshly prepared by diluting original primary or secondary-antibody appropriately in 1% BSA/PBS.

Tissues then incubated with Avidin-Biotin-Complex for 30 min as per manufacturer's instruction, followed by PBS wash for 3 × 3 mins. Staining visualized using DAB (3, 3′-diaminobenzidine tetrahydrochloride-dehydrate)-Kit [BioGenex] by chromogenic-reaction with DAB-solution for 2~10 mins according to manufacturer's instruction. Sections monitored microscopically until optimal-staining was achieved, and the DAB-reaction quenched by dH2O-dilution. Slides counterstained with 60%-Light-Haematoxylin (v/v). Primary-antibody concentrations were as follows: anti-p53 (1:50; Dako, DO-7-clone) [61] and anti-phospho-p53(Ser15) (1:100; Santa-Cruz; sc-101762) or anti-phospho-p53(Ser15) (1:100; Cell Signaling; D4S1H) [62, 63]. For IF, samples exposed to goat anti-rabbit antibodies conjugated to Alexa-Fluor 594 (1/500; Invitrogen) and/or rabbit anti-mouse antibodies conjugated to Alexa-Fluor 488 (1/500; Invitrogen).

### Ultraviolet (UV)-irradiation

Prior to UV-treatment, the cells were starved in RPMI-medium containing 0.1%-FBS for 24 hrs. UV-treatment performed at 50 kJ/m^2^ using Spectrolinker-XL-1000 UV Cross-linker device (Spectromics). Following UV-irradiation, cells incubated for 60 mins in complete-RPMI-medium at 37.5°C, 5%-CO_2_.

### Cell culture, transfection, and Western blot (WB) analysis

*In vitro* analyses were performed by using HCT116 human colon carcinoma cell line deleted for FBXW7 in single (HCT116^FBXW7+/−^) or both alleles (HCT116^FBXW7−/−^) in parallel to the control HCT116^FBXW7+/+^ cell line, which were extensively characterized in our laboratory and others [19, 20, 28]. Transfection of cell lines HEK293 or HCT116 depending on the experiment with DNA plasmids were carried out using Ployethylenimine (PEI) transfection reagent according to the manufacturer's instructions. The protein was extracted using RIPA buffer, as described previously [28, 60]. Eluted proteins in SDS-loading buffer (2×), boiled at 95°C for 4 mins, separated on 8%-SDS-PAGE and transferred to polyvinylidene-fluoride-membranes. p53 mutants were generated with the QuikChange XL Site-Directed Mutagenesis Kit (Stratagene) according to the manufacturer's instructions. Proteins immunoblotted with primary-antibodies; rabbit phospho-p53(Ser15) (1:1000; Cell Signaling), anti-p53 (1:1000; Dako), anti-phospho-p53(Ser15) (1:1000; Santa-Cruz), mouse phospho-p53(Ser18) antibody (1:500; R&D), Fbxw7 (1:500; Bethyl Laboratories), β-Actin (1/10, 000; Abcam), and phospho-p44/42 MAPK (Erk1/2), p44/42 MAPK (Erk1/2), pChk2 (T68), pChk2 (S516), (all 1:1000) were purchased from Cell Signaling Technologies and CKIα from Novus. Secondary-antibodies were HRP-linked goat anti-mouse and goat anti-rabbit antibodies (1/10, 000; Santa-Cruz). Blots were developed using ECL (GE-Healthcare). Experiments repeated on at least two occasions.

### Co-Immunoprecipitation (Co-IP)

HEK293T cells were transfected with plasmids encoding GFP-FBXW7-α [28] and FLAG-p53 (WT) [64]. 30 hrs after transfection, we used 100 μl of anti-FLAG-conjugated agarose beads (Sigma-Aldrich) to immunoprecipitate p53 or anti-GFP antibody-conjugated agarose beads (MBL International) to immunoprecipitate FBXW7 from whole cell extracts of the HEK293T cells using RIPA buffer (150 mM NaCl) with protease inhibitors. After IP, the beads were washed thoroughly with RIPA buffer. Immunoprecipitated proteins were eluted using 2× SDS loading buffer and then boiled at 95°C for 4 mins. Denatured proteins were subsequently separated on 10% SDS PAGE and immunoblotted against anti-GFP and anti-FLAG antibodies as required after transferring to polyvinylidene fluoride membranes as previously described [65]. The experiments were repeated on at least three separate occasions.

### Cytotoxicity/viability and colony forming efficiency assays

Induction of cellular-proliferation through FBXW7-depletion may provide drug-treated cells with survival advantages; therefore prior to treatments, CRC-cells synchronized in G0-phase by serum-free starvation for 18 hrs, and then followed 72 hrs with oxaliplatin treatment (Tocris). To determine the cell-density Sulforhodamine-B (SRB) (Sigma) colorimetric assay was performed. In order to fix the cells, 50 μl of 30%(w/v) cold Trichloroacetic-acid (TCA) added per well and incubated with a final concentration of 10%-TCA for 60 mins at 4°C. After discarding the solution, the plate washed with distilled water and air dried. Then, the fixed cells dyed with 0.4%(w/v) SRB, in 1%-acetic-acid for 20 mins at RT. Following five-washings with 1%-acetic-acid and air-drying, the SRB was dissolved by adding 200 μl of 10 mM-Trizma-base. Lastly, a microplate-reader (Bio-Rad) measured the absorbance at 540 nm-wavelength [66]. Data were elaborated using Microsoft-Office Excel and GraphPad-Prism softwares (http://www.graphpad.com/scientific-software/prism/). Colony forming efficiency assay of HCT116(FBXW7+/+), HCT116(FBXW7−/−) cells treated with 2 different concentrations of oxaliplatin for 72 hours for 200 of seeded cells. Images were taken following colonies fixation and crystal violet staining on day 14 of growth and colonies were counted.

### qRT-PCR analysis

For qRT-PCR analysis [28], total RNA was isolated from CRC cells using TRIZOL reagent (Sigma-Aldrich) and purified using the RNeasy mini kit including DNase (QIAGEN) according to the manufacturer's instructions (Figure S1). cDNA synthesis was performed using Superscript reagents (Invitrogen) according to the manufacturer's instructions. Quantitative real-time PCR was accomplished with SYBR green incorporation (Platinum Quantitative PCR SuperMix-UDG w/ROX; Invitrogen) using an ABI7900HT (Applied Biosystems), and the data were analyzed using SDS 2.3 software (Applied Biosystems). Results were normalized to those obtained with *GAPDH*, and data are presented as fold induction/repression over parental cells. Details of primers used are shown in Table S1. All assays were performed in triplicate at least three times.

### Measuring the level of fluorescence using ImageJ

For quantification of fluorescence intensity, images of IFs were converted into binary images using the ImageJ program (http://rsbweb.nih.gov/ij/download.html) for determining/measuring the level of fluorescence in a selected area. We selected the area of the image with Red-fluorescent (stained with phospho-p53 antibody) first while set measurements for area, integrated density and mean. Basically with selecting “measure” it provide values. Once measurement for red-fluorescent finished, DAPI (blue-fluorescent) were selected. We have also selected area with no fluorescent as background. We have selected 5 areas per slide. We used the following formula to calculate the corrected total cell fluorescence (CTCF). CTCF = Integrated Density – (Area of selected fluorescent × Mean fluorescence of background readings). Then, we have calculated the ratio of average values obtained between p53(Ser15)/DAPI (red/blue) for each condition. We made a graph and showed % of their relative of fluorescence.

### Statistical analysis

Statistical analysis was undertaken using SPSS 15.0 software. All evaluations were done using the unpaired two-tailed Student's *t*-test. For IHC analysis, the chi-square test was used and *p* < 0.05 was considered to indicate a significant difference.

### Supporting information

Supplemental Information contains Supplementary Figure 1, Legend and Supplementary Tables 1, can be found with this article online at *Oncotarget* website.

## SUPPLEMENTARY FIGURE AND TABLE


