# Supplementary material for: FBXW7-mutated colorectal cancer cells exhibit aberrant expression of phosphorylated-p53 at Serine-15
Source: Oncotarget. 2015 Mar 16;6(11):9240–56. doi: 10.18632/oncotarget.3284 (PMC4496214; doi:10.18632/oncotarget.3284)
Supplement: Supplementary file 1 [file oncotarget-06-9240-s001.pdf]

SUPPLEMENTARY FIGURE AND TABLE

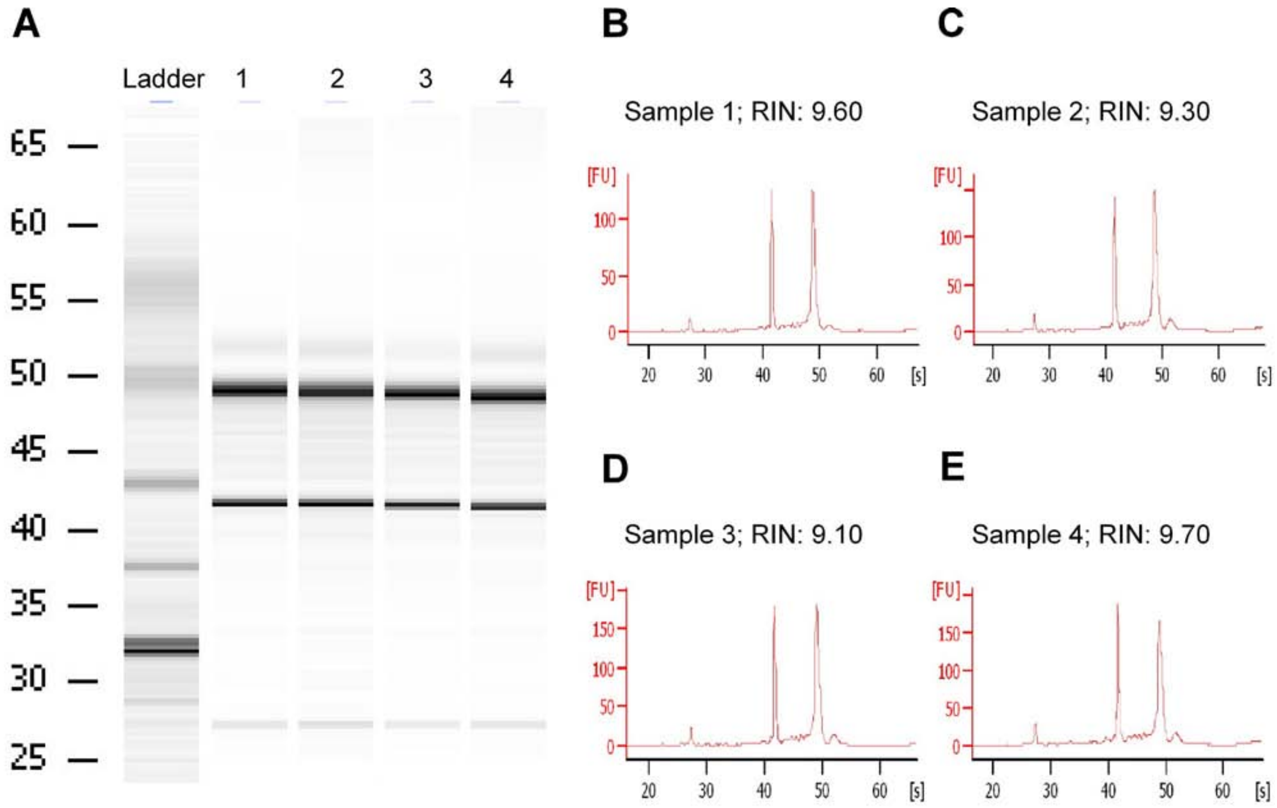

Supplementary Figure S1: A. Representative electropherograms run with Bio-Rad Experion of RNA samples isolated as total RNA from HCT116$^{FBXW7(+/+)}$ (samples 1 and 3) and HCT116$^{FBXW7(-/-)}$ cells (samples 2 and 4). **(B-D)** Representative electropherogram for a good-quality RNA from HCT116$^{FBXW7(+/+)}$ **B and D.** and HCT116$^{FBXW7(-/-)}$ cells **C and E.**

Supplementary Table S1. Primer sequences for the real-time RT-PCR experiments

Gene	primer	sequence (5′-3′)
TP53	Forward	CCCAAGCAATGGATGATTTGA
	Reverse	GGCATTCTGGGAGCTTCATCT
TP63	Forward	AATCTGCTGGTCCATGCTGT
	Reverse	GGAAAACAATGCCCAGACTC
TP73	Forward	GCACCACGTTTGAGCACCTCT
	Reverse	GCAGATTGAACTGGGCCATGA
TP53AIP1	Forward	CCAAGTTCTCTGCTTTC
	Reverse	AGCTGAGCTCAAATGCTGAC
TP53BP2	Forward	TGTGCTTGATGTGTACCTGGAGG
	Reverse	CTAGCAGTAAAGCAAGGGGGTT
GAPDH	Forward	TATTGGGCGCCTGGTCACCA
	Reverse	CCACCTTCTTGATGTCATCA
